# Long-Term Surgical Outcomes of Glaucoma Drainage Implants in Eyes with Preoperative Intraocular Pressure Less than 19 mmHg

**DOI:** 10.1155/2024/6624021

**Published:** 2024-01-24

**Authors:** Shahin Hallaj, Jae-Chiang Wong, Lauren E. Hock, Natasha Nayak Kolomeyer, Aakriti G. Shukla, Michael J. Pro, Marlene R. Moster, Jonathan S. Myers, Reza Razeghinejad, Daniel Lee

**Affiliations:** Glaucoma Service, Wills Eye Hospital, Thomas Jefferson University, Philadelphia, PA 19107, USA

## Abstract

**Background:**

This retrospective review reports on patients who underwent glaucoma drainage implant (GDI) surgery and had baseline intraocular pressure (IOP) of ≤18 mmHg with at least one year of follow-up.

**Methods:**

Clinical data of 67 eyes of 67 patients were collected from patients' charts, and the outcomes of GDI were evaluated until 7 years. GDI failure was defined as IOP reduction of less than 20% from the baseline at two consecutive visits three months after surgery, decline to no light perception, or if additional glaucoma surgery was performed.

**Results:**

The average age was 65.9 ± 13.2 years. Most cases were male (52.2%), White (53.7%), and had primary open-angle glaucoma (62.7%). Forty-four eyes had prior glaucoma surgery (68.6%) and 46 (68.6%) had severe glaucoma. Though postoperative (postop) IOP changes were insignificant, the average postop number of medications dropped from 2.4 ± 1.4 to 1.9 ± 1.2 medications two years after surgery (*p* = 0.0451). Postop complications (23.9%) included GDI exposure (7.5%), inflammation (4.5%), shallow anterior chamber (4.5%), and strabismus (1.5%). Hypotony was observed in 4 eyes (5.9%), none of which developed hypotony maculopathy. The cumulative one-year failure rate was 56.7%, most of which were due to failure to lower IOP.

**Conclusion:**

In patients with baseline IOP ≤18 mmHg who had GDI surgery, though the change in IOP was not statistically significant, the number of medications dropped and visual field progression slowed in a subset of patients with adequate perimetric data. Due to a relatively high rate of complications and limited effectiveness in lowering IOP, GDI should be cautiously used in these eyes.

## 1. Introduction

Glaucoma is a progressive optic neurodegenerative disease that causes visual field (VF) loss and can lead to significant visual impairment [1]. When the patient presents with glaucomatous optic nerve damage and preoperative (preop) intraocular pressure (IOP) < 21 mmHg, managing the condition becomes challenging. In some of these cases, the VF continues to worsen despite clinically controlled IOP. The exact mechanism of disease progression in these cases is not entirely understood.However, several factors, including systemic hypotension, sleep apnea, vascular pathologies, and IOP fluctuations, have been implicated [2–5].

Despite significant advancements in glaucoma treatment, IOP reduction remains the primary therapeutic approach in such cases. This reduction is achieved by using hypotensive glaucoma medications or undergoing glaucoma surgeries. Trabeculectomy, the gold-standard surgical method for glaucoma management, has shown clinical effectiveness in slowing down disease progression in eyes with low to normal baseline IOP in several studies [6–10]. However, this procedure is not without complications and these eyes are prone to postoperative complications, such as hypotony maculopathy and choroidal effusion [11, 12].

In recent years, glaucoma specialists have increasingly turned to glaucoma drainage implant (GDI) surgery as an alternative option for controlling disease progression in refractory glaucoma [13]. Similar to trabeculectomy, GDI surgeries aim to lower IOP by providing an alternative outflow for aqueous humor. Studies, like the primary tube versus trabeculectomy (PTVT) study, have shown that GDI surgeries carry a lower risk of postoperative complications compared to trabeculectomy (29% vs. 41%) [14]. However, GDIshave a higher reported 1-year failure rate compared to the trabeculectomy group (17.3% vs. 7.9%) [15, 16].

Despite the potential advantages of GDI surgeries, there is a lack of comprehensive data on their effectiveness in cases with preop IOP ≤ 18 mmHg. This study aims to investigate the long-term outcomes of GDI surgery in managing glaucoma eyes with preop IOP ≤ 18 mmHg by providing clinical and functional data. Herein, we provide insights into the surgical effectiveness and safety of GDIs in this specific patient population from a tertiary care center, which will inform treatment strategies and clinical decision-making in this population.

## 2. Materials and Methods

This retrospective study reportson adult glaucoma patients who underwent GDI surgery at the glaucoma service of Wills Eye Hospital (a tertiary care center in Philadelphia, USA) between January 1, 2005, and January 1, 2021. Sixty-seven eyes of 67 patients who had an average preop IOP ≤ 18  mmHg, regardless of the number of hypotensive glaucoma medications, were included in the analysis. The patients were required to have at least one year of follow-up to be included in the study.Their clinical data were recorded up to 7 years after surgery. Patients with insufficient pre- and postoperative (postop) data or coexisting ocular conditions that could significantly affect visual acuity, IOP, or glaucoma management were excluded from the study. The following demographic and clinical data were collected: age, sex, race/ethnicity, medical history, medications, surgical history, glaucoma type, and stage, best-corrected visual acuity (BCVA), IOP, lens status, type of GDI, VF data, surgical complications, and need for reoperation. Glaucoma severity was assessed using the Hodapp–Parrish–Anderson criteria [17]. For eyes with missing VF data in the electronic medical records system or picture archiving and communication system (PACS), documented judgments of the physicians based on the criteria were recorded as disease severity. GDIs were considered to have failed if there was a need for IOP-lowering glaucoma reoperation and/or the occurrence of failure criteria. These criteria included progression to no light perception (NLP), less than a 20% IOP reduction, and postop IOP of 18 mmHg or higher at two consecutive visits three months after surgery. The patients' VF mean deviation/defect (MD) were also collected, and the reliability cutoff for false positive and false negative was 33% [18].

### 2.1. Statistical Analysis

Snellen acuities were converted to logMAR values for statistical purposes. Average preop IOP was calculated including IOP measurements of three visits before the surgery. Data and analysis are demonstrated in tables and graphs with colorblind safe colors. GDIs were categorized as valved (i.e., Ahmed FP7 [new world medical, Edison CourtRancho Cucamonga, California, USA]) and nonvalved (including Baerveldt [Abbott Medical Optics, Abbott Park, Illinois, USA], Molteno [Molteno Ophthalmic Limited, Dunedin, New Zealand], and Ahmed valveless Clearpath [ACP, New World Medical, Edison Rancho Cucamonga, California, USA]). Categorical variables were expressed as the frequency and percentage (%) of the total, whereas continuous variables were expressed as average ± standard deviation (SD) in the text and average ± 95% confidence interval in the graphs. Statistical analysis was performed using R Statistical software (version 4.0.5; R Foundation for Statistical Computing, Vienna, Austria) and GraphPad Prism for Windows (version 9.3.1) (GraphPad, La Jolla, CA, USA). The Kolmogorov–Smirnov test was used to identify the normality of the distribution. For numerical variables, *t*-test, Wilcoxon rank-sum, or Wilcoxon signed-rank tests were employed. For categorical variables, *χ*^2^ or or Fisher exact tests were utilized. Linear and binary logistic regression models wereto identify independent predictors of one-year failure. *p* values less than 0.05 were considered significant.

### 2.2. Institutional Review Board Approval

This retrospective cohort study was approved by the Institutional Review Board (IRB) at Wills Eye Hospital (Philadelphia, Pennsylvania, USA) and included patients seen by the Glaucoma Service. The research was conducted in accordance with the Health Insurance Portability and Accountability Act of 1996 and adhered to the tenets of the Declaration of Helsinki.

## 3. Results

### 3.1. Demographics and Preoperative Information

A total of 67 eyes of 67 patients were included in this study. The average age of the patients was 65.9 ± 13.2 years, and 52.2% were male. In terms of race, 53.7% were White, followed by African Americans (26.9%), Hispanics (7.5%), and Asians (2.9%). A majority (62.7%) had primary open-angle glaucoma (POAG). 68.6% had severe glaucoma, followed by moderate (19.4%)) and mild (11.9%). The most common type of implanted GDI was Ahmed FP7 (65.7%), and 34.3% were nonvalved GDIs. The median follow-up period was 26 months, ranging from 12 to 90 months. The baseline average mean defect (assessed by Octopus G tendency-oriented perimetry (TOP)) and mean deviation (assessed by Humphrey Swedish interactive thresholding algorithm (SITA) 24-2) were 15.6 ± 8 decibels (dB) and −16.3 ± 8.5 dB and, respectively. The average preop logMAR BCVA, IOP, and the number of glaucoma medications were 0.9 ± 0.7 [20/150], 13.6 ± 3.4 mmHg, and 2.4 ± 1.4, respectively. 67.1% (*n* = 45) of eyes were pseudophakic, and 41.8% had prior trabeculectomy.  Table 1summarizes the baseline information of the included eyes.

### 3.2. Outcomes

**Figure 1:**
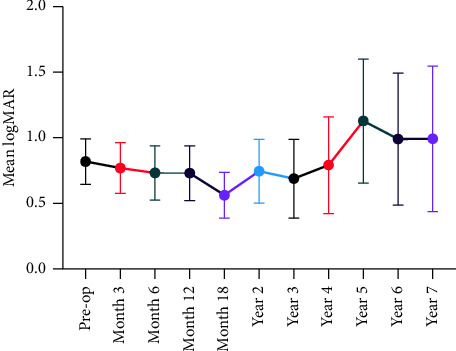
Line graph illustrating the trend in postoperative visual acuity of the eyes, with findings presented as the average ± 95% confidence interval (CI).

The average preop logMAR BCVA of all eyes in this study was 0.9 ± 0.7 [20/150] and ranged from 0 [20/20] to 2.7 (light perception). The follow-up logMAR BCVA ranged from 0 to 3 (NLP), and no significant change was observed in the average logMAR BCVA at each study point compared to the preop visit (*p* = 0.1502). One progressed to NLP three months after the surgery.  Figure 1 illustrates the trend in pre- and postoperative logMAR BC VA.

**Figure 2:**
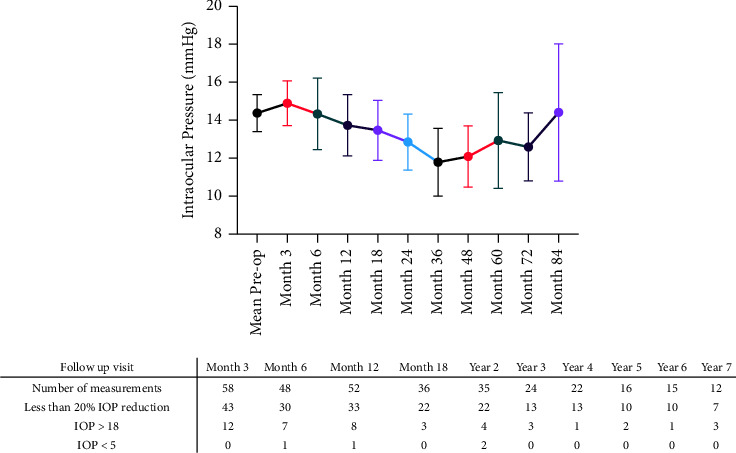
Changes in postoperative intraocular pressure (IOP) among included eyes, with findings illustrated as the average ± 95% confidence interval (CI).

The average preop IOP was 14.6 ± 3.3 mmHg, ranging from 10 to 18 mmHg. No significant difference was found between the average pre- and postop IOP(*p* = 0.0946) (Figure 2). Almost one-third (32.8%, *n* = 22) of the eyes experienced IOP > 18 mmHg at least once during the follow-up period after the first three months (Figure 2). No significant difference was found between the average or median IOPs of valved and nonvalved GDI groups at each study point (*p* > 0.05). Figure 2 demonstrates the trend in postop IOP changes during the follow-up period. The average number of preop prescribed medications was 2.4 ± 1.4 and significantly decreased to 1.9 ± 1.2 after two years of follow-up at a *p* = 0.0451 (Figure 2).

**Figure 3:**
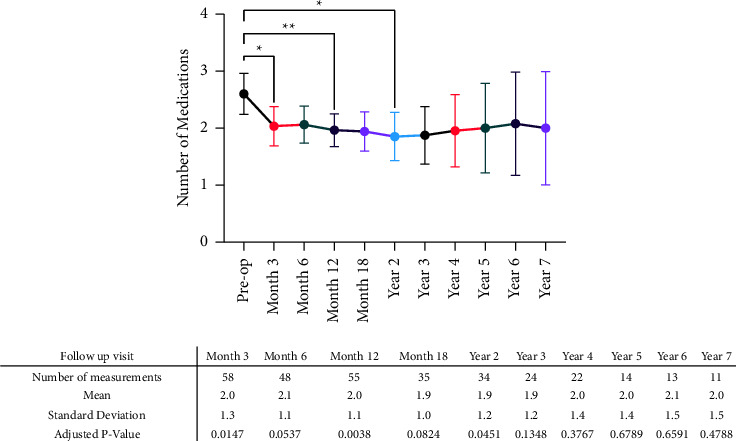
Postoperative changes in the number of prescribed medications among included eyes, with findings presented as the average ± 95% confidence interval (CI). ^*∗*^ and ^*∗∗*^*p* values less than 0.05 and 0.01, respectively.

Among the 67 included eyes, 35 had preop perimetry records in theEMR or PACS, and their average MDs were 15.6 ± 8 dB (*n* = 27) and −16.3 ± 8.5 dB (*n* = 8) as measured by Octopus G TOP and Humphrey SITA 24-2 perimetry, respectively. The average MDs significantly worsened within 2 years before surgery; however, no significant difference was observed in the subsequent follow-up tests. In 16 eyes with at least three visual fields to calculate pre- and postop MD slopes (only G TOP), mean defect slopes were calculated using all available VF data points. The average slopes significantly improved from −2.2 ± 3.1 to −0.2 ± 0.7 dB/year after the surgery at *p* = 0.0176 (Figure 3). This improvement was observed in 81.2% (*n* = 13) of these 16 eyes.

### 3.3. Complications and Failures

Among 67 included eyes, 23.9% (*n* = 16) developed postop complications, including GDI exposure (7.5%, *n* = 5), shallow anterior chamber (4.5%, *n* = 3), and strabismus (1.5%, *n* = 1). Three eyes (4.5%) developed inflammation, two of which were observed in postop month 3 and one was following keratoconjunctivitis in postop year 3. One patient developed endophthalmitis 6 weeks after the GDI surgery (1.5%). Three eyes (4.5%) developed hypotony (IOP < 5mmHg) once during the follow-up, and none had hypotony maculopathy. Nineteen reoperations on 13 eyes (19.4%) were recorded during the follow-up period; only 5 eyes needed further glaucoma surgery to control IOP (3 GDIs and 2 trans-scleral diode laser cyclophotocoagulations). Eight GDI revisions on 5 eyes (7.5%) with GDI erosion were observed, and 3 had more than 1 revisions. GDI explant and insertion of new GDI implant was done in 1 eye due to plate shift (1.5%). Otherwise, GDI removal was done on 2 eyes (2.9%); one due to endophthalmitis and GDI erosion through conjunctiva and the other due to strabismus.

**Figure 4:**
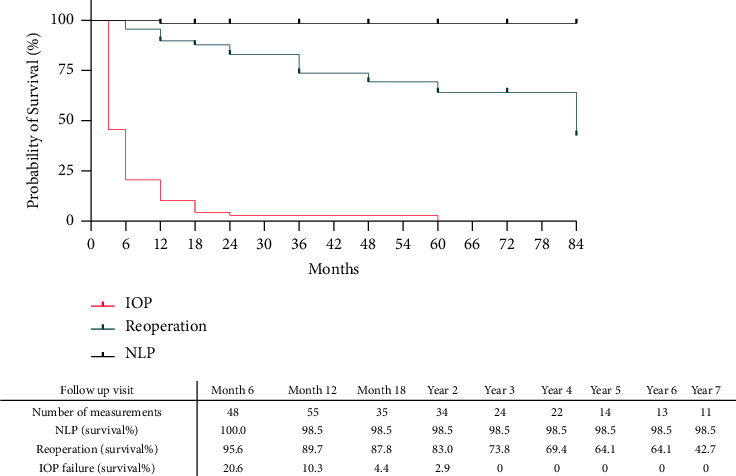
Kaplan–Meier survival curve compares different failure definitions in our population. Censored data points are demonstrated as + in each curve.

The cumulative one-year failure rate was 56.7%. GDI failed to control the IOP in 52.3% of the eyes, 2 eyes (2.9%) needed further glaucoma surgery to control IOP, and 1 eye progressed to NLP (1.5%) within the first year (Figure 4). Though only lower age was significantly correlated with a higher failure rate in the univariable analysis (*β* = −0.258, *R*^2^ = 0.066, *p* = 0.034), no significant independent predictor of one-year failure was found in the multivariable analysis.

## 4. Discussion

In this retrospective cohort study, we evaluated the long-term clinical outcomes of GDI surgeries in eyes with preoperative IOP less than 19 mmHg. To the best of our knowledge, this is the first report on the use of GDIs to manage glaucoma in eyes with the low baseline IOP.

Several previous studies have reported reduced visual field progression following a 30% reduction of IOP [9, 11, 12, 19, 20]. Notably, the Collaborative Normal-Tension Glaucoma Study group demonstrated that lowering IOP, either through medications or trabeculectomy, significantly slowed down the progression rate of visual field loss in a dose-dependent manner [21]. Similarly, Naito et al. reported a significant enhancement of mean deviation (MD) slope following trabeculectomy surgery in eyes with preoperative IOP less than 15 mmHg [22]. In our study, we observed that GDI surgery did not have a significant effect on IOP reduction, unlike the previous reports on trabeculectomy. However, GDI surgery demonstrated significant effectiveness by preserving visual acuity, reducing the number of medications, and slowing down VF loss progression in a subset of patients with adequate data.

In a subanalysis of the PTVT study, Gedde et al. [23–25] compared the outcomes of these two interventions on patients with the baseline IOP <21 mmHg. In their study, GDI failed to control patients' IOP in 63% of the cases after three years. In contrast, our study observed a cumulative one-year failure rate of 56.7%, and the failure rate increased to 86.7% after three years. This difference in failure rates may be attributed to the different baseline characteristics of the study populations, with our study's patients having a significantly lower average baseline IOP compared to theirs.

Regarding postoperative complications, we observed a cumulative complication rate of 23.9% and an overall reoperation rate of 19.4%. In comparison, the PTVT study reported postoperative complications as high as 29% and 34% after one and three years in the GDI group, respectively, compared to 41% and 48% in the trabeculectomy group [16]. However, our study had a significantly higher rate of reoperations due to complications, particularly GDI exposure, which was observed in 7.5% of eyes and recurred in three eyes at subsequent follow-up visits. These differences in complication rates may be related to the unique characteristics of our study population, which consisted of more advanced glaucoma cases with a history of prior hypotensive interventions.

One of the main limitations of our study is the limited number of repetitive visual field tests, which allowed us to evaluate only 16 MD slopes. This limitation raises concerns for unintended selection bias or issues of regression to the mean after rapid progression. Therefore, further investigation with prospective cohort studies may further elucidate our findings. In addition, the majority of the included eyes in our study were at severe glaucoma stages and the surgeries were done by different surgeons with different techniques, which may limit the generalizability of our findings to less advanced cases. However, this is the first study providing data on the use of GDIs in this patient population. These data can be used to inform clinical decision-making for this specific population.

In conclusion, GDI surgeries may be used in patients with lower preop IOP to reduce the number of required hypotensive medications. However, our study suggests that GDIs did not lead to significant IOP reduction and were associated with a considerably high complication rate. Therefore, careful consideration should be exercised when using GDIs in eyes with lower preop IOP. Further research may help further elucidate the findings of this study and the role of GDIs in managing glaucoma in these cases.

## Figures and Tables

**Table 1:** Demographic profile and clinical characteristics of the included glaucoma patients.

	*N* (%)	Average ± SD
Gender		
Male	35 (52.2%)	
Female	32 (47.8%)	
Race		
White	36 (53.7%)	
African American	18 (26.9%)	
Hispanic	5 (7.5%)	
Asian	3 (4.4%)	
Declined to specify	5 (7.5%)	
Glaucoma type		
POAG^*∗*^	42 (62.6%)	
Secondary to other eye diseases^*∗∗*^	11 (16.4%)	
Angle closure glaucoma	5 (7.5%)	
Pseudoexfoliative (PEX)	5 (7.5%)	
Normal-tension glaucoma	3 (4.5%)	
Other specified glaucoma^*∗∗∗*^	1 (1.5%)	
Glaucoma severity		
Severe	46 (68.6%)	
Moderate	13 (19.4%)	
Mild	8 (11.9%)	
Type of tube		
Valved	44 (65.7%)	
Nonvalved	23 (34.3%)	
Visual fields		
Octopus G TOP (mean defect dB)	27	15.6 ± 8
Humphrey SITA 24-2 (mean deviation dB)	8	−16.3 ± 8.5
Visual acuity (logMAR)		0.9 ± 0.7
IOP (mm·Hg)		14.6 ± 3.3
Number of medications		2.4 ± 1.4
Lens status		
Pseudophakic	45 (67.1%)	
Phakic	17 (25.3%)	
Aphakic	5 (7.6%)	
Eye		
OS	30 (44.8%)	
OD	37 (55.2%)	
Past ocular procedures		
Phacoemulsification and IOL	40 (59.7%)	
Trabeculectomy	28 (41.8%)	
Selective laser trabeculoplasty	23 (33.8%)	
Corneal surgery	14 (20.6%)	
Minimally invasive glaucoma surgery	6 (8.8%)	
Argon laser trabeculoplasty	5 (7.4%)	
Vitreoretinal surgery	5 (7.4%)	
Peripheral iridotomy	4 (5.9%)	
Diode cyclophotocoagulation	2 (2.9%)	

Summary of the cases; ^*∗*^primary open-angle glaucoma, including open-angle with borderline findings; ^*∗∗*^secondary glaucoma (i.e., after trauma, steroids, Sturge–Weber, neovascular glaucoma, and inflammatory); others (e.g., congenital); ^*∗∗∗*^following penetrating keratoplasty. TOP: tendency-oriented perimetry; SITA: Swedish interactive thresholding algorithm; IOP: intraocular pressure; IOL: intraocular lens.

## Data Availability

The data used to support the findings of this study are available from the corresponding author upon reasonable request.

## References

[1] Deokule S., Weinreb R. N. (2008). Relationships among systemic blood pressure, intraocular pressure, and open-angle glaucoma. *Canadian Journal of Ophthalmology*.

[2] Hallaj S., Mirza-Aghazadeh-Attari M., Arasteh A., Ghorbani A., Lee D., Jadidi-Niaragh F. (2021). Adenosine: the common target between cancer immunotherapy and glaucoma in the eye. *Life Sciences*.

[3] Leggewie B., Gouveris H., Bahr K. (2022). A narrative review of the association between obstructive sleep apnea and glaucoma in adults. *International Journal of Molecular Sciences*.

[4] Leidl M. C., Choi C. J., Syed Z. A., Melki S. A. (2014). Intraocular pressure fluctuation and glaucoma progression: what do we know?. *British Journal of Ophthalmology*.

[5] Nicolela M. T. (2008). Clinical clues of vascular dysregulation and its association with glaucoma. *Canadian Journal of Ophthalmology*.

[6] Aoyama A., Ishida K., Sawada A., Yamamoto T. (2010). Target intraocular pressure for stability of visual field loss progression in normal-tension glaucoma. *Japanese Journal of Ophthalmology*.

[7] de Jong N., Greve E. L., Hoyng P. F. J., Geijssen H. C. (1989). Results of a filtering procedure in low tension glaucoma. *International Ophthalmology*.

[8] Jayaram H., Strouthidis N. G., Kamal D. S. (2016). Trabeculectomy for normal tension glaucoma: outcomes using the Moorfields Safer Surgery technique. *British Journal of Ophthalmology*.

[9] Nakajima K., Sakata R., Ueda K. (2021). Central visual field change after fornix-based trabeculectomy in Japanese normal-tension glaucoma patients managed under 15 mmHg. *Graefe’s Archive for Clinical and Experimental Ophthalmology*.

[10] Shigeeda T., Tomidokoro A., Araie M., Koseki N., Yamamoto S. (2002). Long-term follow-up of visual field progression after trabeculectomy in progressive normal-tension glaucoma. *Ophthalmology*.

[11] Anderson D. R., Drance S. M., Schulzer M. (1998). Comparison of glaucomatous progression between untreated patients with normal-tension glaucoma and patients with therapeutically reduced intraocular pressures. *American Journal of Ophthalmology*.

[12] Razeghinejad M. R., Lee D. (2019). Managing normal tension glaucoma by lowering the intraocular pressure. *Survey of Ophthalmology*.

[13] Myers J. S., Lamrani R., Hallaj S., Lee D., Wong J. C. (2023). 10-Year clinical outcomes of tube shunt surgery at a tertiary care center. *American Journal of Ophthalmology*.

[14] Gedde S. J., Chen P. P., Heuer D. K. (2018a). The primary tube versus trabeculectomy study: methodology of a multicenter randomized clinical trial comparing tube shunt surgery and trabeculectomy with mitomycin C. *Ophthalmology*.

[15] Gedde S., Heuer D. K., Parrish R. K. (2010). Review of results from the tube versus trabeculectomy study. *Current Opinion in Ophthalmology*.

[16] Gedde S. J., Herndon L. W., Brandt J. D. (2012). Postoperative complications in the Tube versus Trabeculectomy (TVT) study during five years of follow-up. *American Journal of Ophthalmology*.

[17] Anderson D. R., Hodapp E., Parrish R. K. (1993). *Clinical Decisions in Glaucoma*.

[18] Berezina T. L., Khouri A. S., Winship M. D., Fechtner R. D. (2012). Visual field and ocular safety during short-term vigabatrin treatment in cocaine abusers. *American Journal of Ophthalmology*.

[19] Iverson S. M., Schultz S. K., Shi W., Feuer W. J., Greenfield D. S. (2016). Effectiveness of single-digit IOP targets on decreasing global and localized visual field progression after filtration surgery in eyes with progressive normal-tension glaucoma. *Journal of Glaucoma*.

[20] Wang S. Y., Singh K. (2020). Management of the glaucoma patient progressing at low normal intraocular pressure. *Current Opinion in Ophthalmology*.

[21] Oie S., Ishida K., Yamamoto T. (2017). Impact of intraocular pressure reduction on visual field progression in normal-tension glaucoma followed up over 15 years. *Japanese Journal of Ophthalmology*.

[22] Naito T., Fujiwara M., Miki T. (2017). Effect of trabeculectomy on visual field progression in Japanese progressive normal-tension glaucoma with intraocular pressure < 15 mmHg. *PLoS One*.

[23] Gedde S. J., Feuer W. J., Shi W. (2018b). Treatment outcomes in the primary tube versus trabeculectomy study after 1 Year of follow-up. *Ophthalmology*.

[24] Gedde S. J., Feuer W. J., Lim K. S. (2020). Treatment outcomes in the primary tube versus trabeculectomy study after 3 Years of follow-up. *Ophthalmology*.

[25] Gedde S. J., Feuer W. J., Chen P. P., Heuer D. K., Singh K., Wright M. M. (2021). Comparing treatment outcomes from the tube versus trabeculectomy and primary tube versus trabeculectomy studies. *Ophthalmology*.

